# Advances in CAR-T-cell therapy in T-cell malignancies

**DOI:** 10.1186/s13045-024-01568-z

**Published:** 2024-06-24

**Authors:** Rubing Zheng, Xiaojian Zhu, Yi Xiao

**Affiliations:** Tongji Hospital, Tongji Medical College, Huazhong University of Science and Technology, Wuhan, 430030 China

**Keywords:** T-cell malignancies, CAR-T-cell therapy, Target antigens, Challenges, Research advances

## Abstract

Significant advances have been made in chimeric antigen receptor T (CAR-T)-cell therapy for the treatment of recurrent or refractory B-cell hematologic malignancies. However, CAR-T-cell therapy has not yet achieved comparable success in the management of aggressive T-cell malignancies. This article reviews the challenges of CAR-T-cell therapy in treating T-cell malignancies and summarizes the progress of preclinical and clinical studies in this area. We present an analysis of clinical trials of CAR-T-cell therapies for the treatment of T-cell malignancies grouped by target antigen classification. Moreover, this review focuses on the major challenges encountered by CAR-T-cell therapies, including the nonspecific killing due to T-cell target antigen sharing and contamination with cell products during preparation. This review discusses strategies to overcome these challenges, presenting novel therapeutic approaches that could enhance the efficacy and applicability of CAR-T-cell therapy in the treatment of T-cell malignancies. These ideas and strategies provide important information for future studies to promote the further development and application of CAR-T-cell therapy in this field.

## Background

Over the past few years, CAR-T-cell therapy has emerged as a major breakthrough in the treatment of relapsed/refractory (R/R) hematologic malignancies. CD19 is widely expressed in the early to mature stages of B cells, encompassing both malignant and normal B cells. BCMA expression is elevated in B-cell lymphomas but is reduced in normal tissues. The U.S. Food and Drug Administration (FDA) has approved several CAR-T-cell products targeting CD19 and BCMA due to their significant therapeutic efficacy against specific B-cell malignancies, including diffuse large B-cell lymphoma, B-cell acute lymphoblastic leukemia, follicular lymphoma, and multiple myeloma [1]. Treatment-induced adverse effects, such as hypogammaglobulinemia, are effectively managed with intravenous immunoglobulin (IVIG). These findings highlight the availability and safety of existing targets for the treatment of B-cell lymphoma. Unlike B-cell malignancies, T-cell malignancies constitute a highly heterogeneous group of diseases with significant diversity at the molecular and cellular levels and varied clinical manifestations, disease progression rates, and responses to therapy, posing major therapeutic challenges. As the biology of these diseases is better characterized, new therapeutic targets and strategies are being developed to improve therapeutic efficacy and patient prognosis. However, when applying CAR-T-cell therapy to treat malignancies, researchers face multiple challenges, particularly the lack of specific target antigens, nonspecific killing of normal T cells, and interactions between CAR-T cells, which can lead to severe immunodeficiency and hematopoietic toxicity, limiting the application and efficacy of CAR-T-cell therapy in the treatment of T-cell malignancies. Thus, although research advances offer hope for future therapies, challenges remain in the treatment of this highly heterogeneous and refractory disease. This review aims to explore the challenges of applying CAR-T-cell therapy in the treatment of T-cell malignancies by reviewing relevant preclinical studies and clinical trials categorized by target antigens to guide future research in overcoming these challenges and enhancing the efficacy and applicability of CAR-T-cell therapies for treating T-cell malignancies.

## Overview of T-cell malignancies

T-cell malignancies encompass a heterogeneous array of diseases that exhibit malfunction and clonal expansion at different stages of T-cell development. Depending on the origin of the tumor, the tissues it affects, and its clinical manifestations, these diseases can be classified into several subtypes, each of which reveals the biological characteristics of the disease and the behavior of clonal T cells, providing an important basis for classification for diagnosis and research.

Peripheral T-cell lymphoma (PTCL) is a highly heterogeneous group of malignant lymphomas derived from T cells at different developmental stages and is commonly found in the lymph nodes, spleen and other organs. The World Health Organization has classified it into approximately 30 subtypes [2]. Common subtypes include PTCL, not otherwise specified (PTCL-NOS), angioimmunoblastic T-cell lymphoma (AITL), and mesenchymal anaplastic large cell lymphoma (ALCL). In China, the incidence of PTCL is significantly higher than that in North America and Western European countries, accounting for 23–27% of all non-Hodgkin’s lymphomas (NHLs), reflecting significant geographic variation and disease heterogeneity [3]. Treating PTCL is particularly challenging, mainly due to the lack of clear molecular markers and uniform morphological features. Traditionally, treatments have included chemotherapy and stem cell transplantation; however, many patients progress, with a median survival of only 5–8 months and a five-year survival rate of merely 38.5% [4]. Recently, the use of targeted agents, particularly CAR-T-cell therapy, has translated the success achieved in treating B-cell lymphoma into accelerated research on T-cell lymphoma.

Cutaneous T-cell lymphomas (CTCLs) are a heterogeneous group of T-cell malignancies that specifically affect the skin and present as plaques, papules, or masses, with mycosis fungoides (MF) and Sézary syndrome (SS) as the most common subtypes, accounting for 60–70% of all CTCL cases [5]. These tumor cells primarily accumulate in the skin but may metastasize to the blood and other organs in advanced stages, often leading to resistance to chemotherapy. Strategies for treating CTCL depend on the stage of the disease: localized treatment is mostly used in the early stages, while systemic therapy is required in the advanced stages. However, malignant T cells in MF/SS exhibit resistance to conventional chemotherapy, resulting in a poor prognosis due to the absence of standardized treatments and early inertia in disease management [6]. The treatment of advanced CTCL is particularly challenging, and the primary cause of mortality is infection stemming from T-cell immunodeficiency.

T-lymphocytic leukemia/lymphoma (T-ALL/LBL) is a malignancy originating from T-cell precursors, which, based on the proportion of malignant cells in the bone marrow, manifests either as acute T-lymphoblastic leukemia (T-ALL) or T-cell lymphoblastic lymphoma (T-LBL) [7]. These diseases mainly affect children and young adults and are characterized by the abnormal proliferation of T cells, which causes the rapid progression of hematological disorders and lymphoid tissue enlargement. The disease is divided into subtypes based on immunophenotypic, molecular genetic and clinical features, and these subtypes differ significantly in the treatment response and prognosis. Conventional treatments, including chemotherapy, stem cell transplantation, and targeted therapies, are typically unsatisfactory for patients with R/R T-ALL/LBL, particularly those with T-LBL, who experience high relapse rates and a five-year survival rate of only 30–50% [8].

Adult T-cell leukemia/lymphoma (ATLL) is caused by human T-cell leukemia virus type 1 (HTLV-1) infection and primarily affects adults in HTLV-1 endemic areas. Natural killer (NK)/T-cell lymphoma (NKTCL) is a rare malignant lymphoma closely linked to Epstein-Barr virus (EBV) infection, predominantly arising from NK cells or T cells with NK-like characteristics. Predominantly occurring in Asia and Latin America, this malignancy is characterized by a poor prognosis.

Compared to B-cell malignancies, T-cell diseases are less well understood and generally have a poorer prognosis. Despite improvements in chemotherapeutic regimens, the therapeutic efficacy against most R/R PTCL subtypes and T-ALL/LBL is limited, CTCL has a variable prognosis, and the treatment of ATLL and NKTCL remains challenging. Immunotherapies have revolutionized the landscape of cancer treatment, yet patients with T-cell disorders have shown limited responses. In this context, CAR-T-cell therapy, an innovative form of immunotherapy, presents new possibilities for treating T-cell malignancies by specifically engineering a patient’s T cells to recognize and eliminate cancer cells. However, the development of CAR-T-cell therapies targeting T-cell malignancies has encountered significant challenges, primarily due to the frequent lack of specific surface antigens on these malignant cells. Researchers have identified several potential targets, including CD5, CD7, CD30, and TRBC, suggesting promising avenues for CAR-T-cell therapy. Due to the high heterogeneity of T-cell malignancies and the potential for normal T cells to also express these targets, the development of such therapies necessitates a meticulous design and rigorous safety assessments to ensure the effective targeting of tumor cells with a minimal impact on the normal immune system. Researchers continue to explore new targets to optimize antigen binding and enhance the efficacy of CAR-T-cell therapies, an approach anticipated to play a major role in the future treatment of T-cell malignancies.

## Exploration of CAR-T-cell therapy targets in T-cell malignancies

**Fig. 1:**
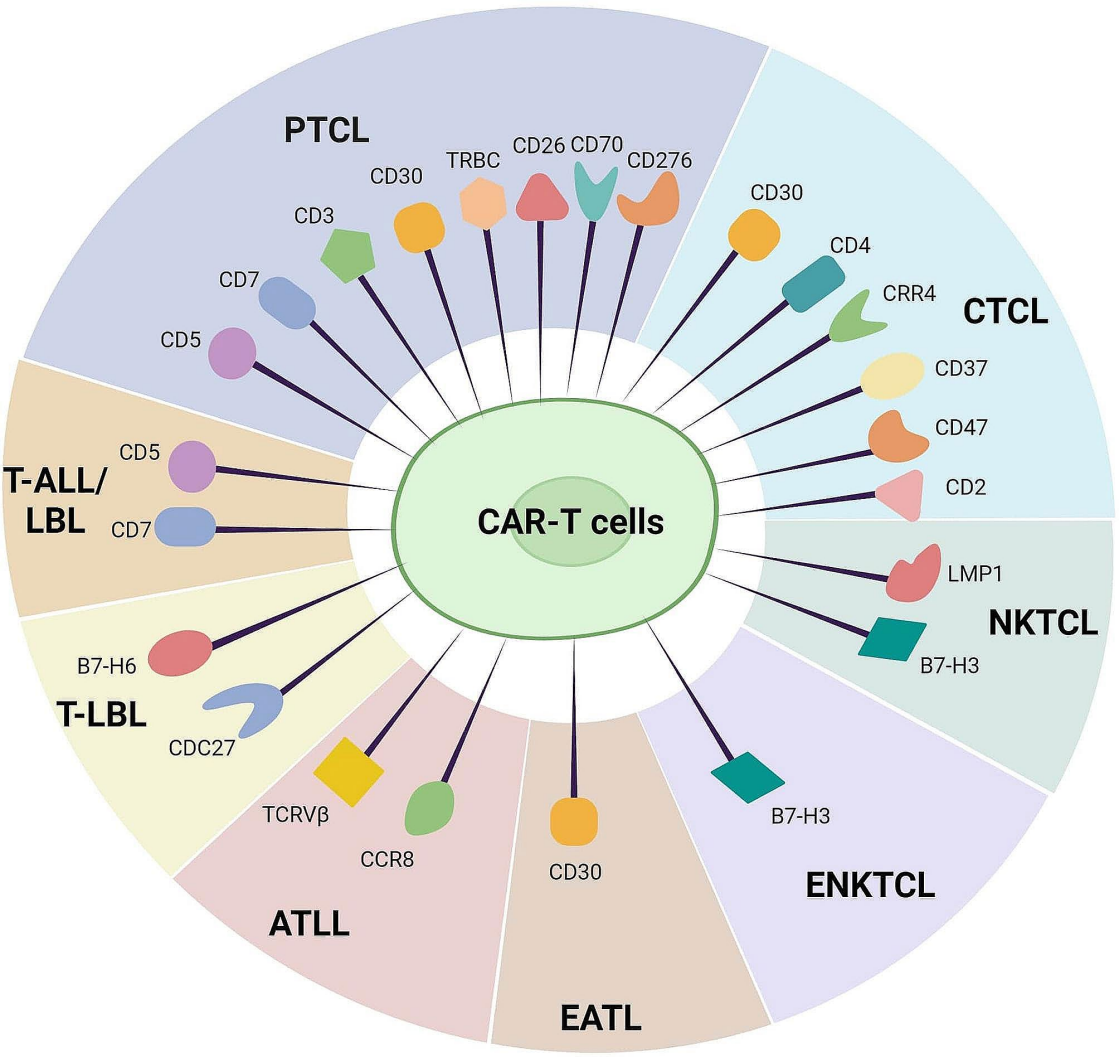
Therapeutic targets of CAR-T-cell therapy for T-cell malignancies. PTCL, peripheral T-cell lymphoma; CTCL, cutaneous T-cell lymphoma; T-ALL/LBL, T-lymphocytic leukemia/lymphoma; T-LBL, T-cell lymphoblastic lymphoma; ATLL, adult T-cell leukemia/lymphoma; NKTCL, NK/T-cell lymphoma; ENKTCL, extranodal nasal NK/T-cell lymphoma; EATL, enteropathy-associated T-cell lymphoma. Created with BioRender.com

In this section, we provide a comprehensive examination of potential targets for CAR-T-cell therapy for the treatment of T-cell malignancies. We explored 17 distinct targets, each at varying stages of research and clinical validation. These targets include CD5, CD7, and TRBC, which have undergone extensive validation through numerous preclinical studies and clinical trials; targets such as CD2 and CD3, which are in the preclinical research stage; and targets such as CD4 and CD30, which are currently being investigated in clinical trials to further validate their therapeutic effects. This research underscores the potential and challenges of CAR-T-cell therapy for treating T-cell malignancies, providing a crucial scientific foundation for future therapeutic developments and applications. Figure 1 illustrates potential targets for CAR-T-cell therapy in various subtypes of T-cell malignancies, and a detailed target analysis is presented in Table 1.

**Table 1:** Analysis of key targets for CAR-T-cell therapy for T-cell malignancies

Targets	Properties	T-cell malignancies	Research phases	Nontarget cells/Tissues	Challenges
CD5	Glycoprotein on T and B-1a cells, crucial for TCR signaling, aberrantly expressed in some PTCLs. Regulates T-cell proliferation in T-ALL/LBL and enhances tumor cytotoxicity.	PTCL, T-ALL/LBL	Preclinical and clinical studies [9–15]	Normal T cells and some B cells	Fratricide and Suicide
CD7	Expressed on T/NK cells, high expression in T-ALL and some PTCLs.	PTCL, T-ALL/LBL	Preclinical and clinical studies (NCT04004637 and NCT04033302) [16–20]	Normal T cells, some NK cells and hematopoietic stem cells	Fratricide and Suicide
TRBC	Expressed exclusively in T cells, distinguishes between TRBC1/TRBC2 in healthy/malignant T cells.	PTCL	Clinical studies [21–23]	Normal T cells	Fratricide and Suicide
CD2	Expressed in T-cell malignancies, potential therapeutic target.	CTCL	Preclinical studies [24]	Most normal T cells, NK cells and certain cells in hematopoietic tissues	Fratricide and Suicide
CD3	Surface protein complex essential for T-cell activation.	PTCL	Preclinical studies [25, 26]	Normal T cells	Fratricide and Suicide
CD4	Marker of T cells; expansion in MF/SS CTCL subtypes indicates its potential as a therapeutic target.	CTCL	Preclinical and clinical studies (NCT03829540, NCT04219319, and NCT04162340) [27, 28]	Helper T cells and certain cells in hematopoietic tissues	Fratricide and Suicide
CD30	TNF receptor family member, expressed in certain activated T/B lymphocytes, high expression in PTCL/ALCL.	PTCL, CTCL, EATL	Preclinical and clinical studies (NCT02917083, NCT01316146, NCT03049449, and NCT02690545) [29–33]	Activated B cells and T cells	Suicide
CCR4	G protein-coupled receptor, regulates T-cell migration/function, expressed in T-cell malignancies.	CTCL	Preclinical studies [34, 35]	Regulatory T cells	Suicide
CD26	Membrane glycoprotein predominantly found on CD4 + T cells, associated with activation/signaling.	PTCL	Preclinical studies [36]	A variety of normal cells, including those of the digestive system and lungs	Suicide
CD70	TNF ligand family member, expressed in activated T cells, leukemias, and lymphomas.	PTCL	Preclinical studies [37]	Activated B cells, T cells	Suicide
CD276 (B7–H3)	B7 family member, expressed in solid tumors and ALCL, linked to immune evasion.	PTCL, NKTCL, ENKTCL	Preclinical studies [38]	/	Suicide
CD37	Transmembrane 4 superfamily member, expressed on mature B cells and some T/NK cells.	CTCL	Clinical studies [39, 40]	Normal B cells	Neither
CD47	Integrin-associated protein, high expression in SS, correlates negatively with survival.	CTCL	Clinical studies [41]	Hematopoietic and nonhematopoietic cells	Neither
B7–H6	Novel costimulatory molecule in T-LBL, linked to poor prognostic factors.	T-ALL/LBL	Preclinical studies [42]	/	Neither
CDC27	Cell cycle-associated protein, overexpressed in T-LBL, linked to disease progression and reduced survival.	T-ALL/LBL	Preclinical studies [43, 44]	/	Neither
TCRVβ	Beta chain of the TCR α/β heterodimer, crucial for T-cell immune responses, expressed on normal/malignant T cells.	ATLL	Preclinical studies [45]	/	Neither
CCR8	G protein-coupled receptor, upregulated in ATLL Treg cells, its blockade boosts anti-tumor immunity.	ATLL	Preclinical studies [46]	/	Neither

Abbreviations: ATLL: adult T-cell leukemia/lymphoma; CTCL: cutaneous T-cell lymphoma; LBL: lymphoblastic lymphoma; MF: mycosis fungoides; NK: natural killer; PTCL: peripheral T-cell lymphoma; T-ALL: T-cell acute lymphoblastic leukemia; TCR: T-cell receptor; TCRVβ: T-cell receptor variable beta; TNF: tumor necrosis factor; TRBC: T-cell receptor β-chain; Tregs: regulatory T cells; SS: Sézary syndrome

### CD5

CD5, a transmembrane glycoprotein belonging to the cysteine-rich scavenger receptor family, is widely expressed in thymocytes, peripheral T lymphocytes, and B-1a cell subpopulations and plays a negative regulatory role in T-cell receptor (TCR) signaling during normal T-cell activation primarily to reduce autoreactive T-cell function. This finding suggests the potential therapeutic value of CD5 in treating T-cell malignancies. In a phase I clinical trial, fourteen patients with T-cell lymphoma underwent treatment with combination therapy consisting of a monoclonal antibody targeting CD5 and an immunotoxin. The outcomes revealed that four of these patients achieved partial remission, with durations ranging from three to eight months. Notably, all observed side effects were reversible, underscoring the high specificity and minimal adverse impact of this therapy on patients with this form of malignancy [47]. Consequently, CD5 expression in T-cell malignancies positions it as a primary candidate for development into a pan-T-cell antigen CAR-T-cell target.

Although CD5 is expressed in normal T cells, potentially leading to self-incompatibility and impaired T-cell regeneration, CD5 CAR-T cells are designed to minimize the impact on nonmalignant cells by preferentially targeting malignant T cells and downregulating CD5 to prevent CAR-mediated self-destruction [48]. Advances in genome editing technologies have provided new tools for CD5-targeting strategies. Using CRISPR-Cas9 technology, researchers have determined that modulating CD5 expression does not adversely affect T-cell proliferation; however, it enhances the toxicity of these cells toward tumor cells [9]. Raikar et al. [10] employed CRISPR-Cas9 technology to knock down CD5 in primary T cells, followed by transduction with CD5-CAR, enhancing both the surface expression of CAR and its antitumor efficacy while reducing self-activation. Similarly, Angelos et al. [11] developed a novel Senza5 CAR-T-cell product, which, by knocking down autologous CD5, showed high specificity and low off-target risks, maintained pathogen responsiveness, and has been utilized in phase I trials for treating R/R CD5-positive T-cell lymphomas. Recently, Hill L.C. and his team [49] introduced a novel CD5 CAR-T-cell platform that minimizes damage to normal T cells by reducing CD5 protein levels but maintains robust cytotoxicity against CD5-positive malignancies. In the inaugural human trial (NCT0308190), the team prepared second-generation autologous CD5 CAR-T cells for treating nine individuals with R/R T-cell malignancies. The overall response rate was 44%, including 2 patients who achieved complete remission. No serious or irreversible adverse events were observed during treatment; however, three patients developed prolonged severe cytopenia, one of whom met the dose-limiting toxicity criteria. These findings indicate that CD5 CAR-T-cell therapy can safely elicit clinical responses in patients with CD5-positive T-cell lymphomas without compromising endogenous T cells or increasing the infection risk, although further validation with more patients and extended follow-up is needed.

Further improving the CD5-targeting efficacy, Mamonkin M et al.‘s study [12] showed that CD5-targeted CAR-T cells with the CD28 costimulatory domain effectively reduced CD5 surface expression, diminished autophagy triggered by self-targeting, and exhibited significant cytotoxicity in vitro and in vivo in a T-ALL model. Subsequent research revealed that CAR-T cells incorporating the 4-1BB costimulatory domain significantly reduced self-incompatibility. Additionally, the use of the Tet-OFF expression system has been shown to mitigate self-incompatibility during in vitro expansion, substantially improving survival rates in vivo [13]. These technological advances provide potential solutions to the problem of T-cell regenerative disorders and reveal their diverse applications in the treatment of complex diseases.

Moreover, NK-92 cells, a CD5-negative NK cell line, were utilized to enhance cytotoxicity against T-cell leukemia via CD5-CAR, which significantly improved survival in a T-ALL xenograft model. Conversely, CD5-CAR-modified NK-92 cells generated using the NK-specific costimulatory domain 2B4 outperformed those generated with the CD28-41BB-CAR in both in vitro and in vivo studies [50]. In the MAGENTA study (NCT03081910) [14], five patients with CD5-positive R/R T-ALL/LBL received specific second-generation CD5 CAR-T cells; two achieved complete remission (CR), and one patient with extensive AITL underwent hematopoietic stem cell transplantation (HSCT) after a second infusion due to new lesion development, maintaining CR for six months posttransplantation.

Central nervous system (CNS) invasion by T-cell leukemia/lymphoma often leads to a poor prognosis, and traditional preventive measures such as radiotherapy and intrathecal chemotherapy are limited due to their adverse effects [51]. CAR-T-cell therapies can breach the blood‒brain barrier, representing a novel treatment option for T-cell malignancies with CNS involvement. IL-15, a cytokine, promotes the proliferation, survival, and antitumor activity of T cells. Leveraging these properties, Feng et al. [15] developed a novel CD5-IL15/IL15sushi CAR-T-cell therapy that enhances malignant T-cell clearance by secreting a soluble IL-15 protein linked to the sushi domain of the IL-15 receptor α while preserving normal T-cell function. This approach proved to be highly effective in killing CD5-positive cell lines in vitro, and in early clinical trials, it rapidly cleared lymphoblastoid cells from the cerebrospinal fluid of patients with recurrent T-LBL involving the CNS, significantly improving symptoms and sparing normal CD5 + T cells; thus, this novel CAR-T-cell therapy is a safe and effective treatment option.

### CD7

CD7 is a transmembrane glycoprotein ubiquitously expressed on most T cells and NK cells and is particularly highly expressed in T-ALL and some PTCL subtypes, making it a key target for immunotherapy for T-cell malignancies. Modified immunotoxins and monoclonal antibodies exhibit specific binding and endocytosis when targeting CD7 molecules, effectively inhibiting the growth of CD7-positive Jurkat and CEM cell lines, as well as T-ALL patient samples. These findings underscore the specificity and efficacy of targeting T-cell malignancies with CD7 in both preclinical and clinical studies [52]. Studies using CD7 knockout mice, which involve normal lymphoid organs and immune responses, indicate that CD7 is dispensable for T/NK cell development and function [53]. Unlike that of CD5, the internalization of CD7 on the surface of T cells after CAR expression is incomplete, leading to extensive self-attack by CD7 CAR-T cells. This process presents a challenge in PTCL treatment due to the limited expansion capacity of CD7 CAR-T cells, given that normal T cells also express CD7. Therefore, Gomes-Silva et al. [20] employed CRISPR-Cas9 technology to disrupt the CD7 gene before CAR expression, minimizing self-attack in T cells while maintaining their cytotoxicity against tumors. This approach not only improved the safety and efficacy of CAR-T-cell therapy but also exerted protective effects on a T-ALL mouse xenograft model.

The initial human trial utilizing donor-derived CD7 CAR-T cells to treat R/R T-ALL, which was conducted by Pan J and his team in a single-center phase I clinical trial [54], involved infusing 20 patients with these cells. Remarkably, 90% (18/20) achieved CR, with only grade 1–2 cytokine release syndrome (CRS) observed. This study revealed that while treatment led to the depletion of normal CD7-positive T cells, it expanded CD7-negative T cells, potentially mitigating treatment-associated T-cell immunodeficiency. By observing a high CR rate and a well-managed safety profile, this study confirmed the feasibility of CD7 CAR-T-cell therapy. Additionally, a multicenter phase II trial (NCT04689659) involving donor-derived CD7 CAR-T cells is currently underway. In two subsequent clinical trials (ChiCTR1900025311 and ISRCTN19144142) [55], investigators introduced a novel ‘off-frame’ allogeneic CAR-T-cell therapy, GC027, targeting CD7 in patients with R/R T-ALL. CAR-T cells quickly expanded, effectively clearing CD7-positive T lymphoblasts from peripheral blood, bone marrow, and cerebrospinal fluid. Both participants achieved sustained CR, although they experienced grade 3 CRS. Notably, no instances of graft-versus-host disease (GvHD) occurred. These two clinical trials demonstrated that GC027, an innovative CD7-targeted “off-frame” allogeneic CAR-T-cell regimen, may be an effective and durable treatment option for patients with R/R T-ALL. In another phase I trial, Hu et al.‘s [56] team administered RD13-01, a genetically modified allogeneic CAR-T-cell therapy targeting CD7, to 12 patients. Twenty-eight days after treatment, 81.8% (9/11) of the patients exhibited an objective therapeutic response, achieving a CR rate of 63.6% (7/11), with no serious adverse effects reported. Similarly, Chen et al. [16] conducted a phase I clinical trial (ChiCTR2200058969) with donor-derived CD7 CAR-T cells and achieved 100% optimal CR in seven patients with R/R T-ALL/LBL. Notably, through single-cell sequencing analysis, they also highlighted the potential role of CD7 CAR-T cells in immune reconstitution, opening new research avenues for treating highly aggressive CD7-positive malignant T-cell tumors. These preliminary results suggest that donor-derived CD7 CAR-T-cell therapy shows encouraging safety and efficacy.

In addition to the application of CAR-T-cell therapy alone, researchers have explored the combined strategy of bridging HSCT following CAR-T-cell therapy. Notably, Yongxian Hu and his team enrolled 10 patients with R/R CD7-positive ALL/LBL in clinical trials (NCT04599556 and NCT04538599) [57]. All patients achieved CR following CD7 CAR-T-cell therapy but had incomplete hematologic recovery and subsequently received haploidentical HSCT. The results indicated that all patients achieved CR, 8 patients achieved complete donor chimerism, and only 3 patients developed grade 2 HSCT-associated acute GvHD. This strategy offers a viable treatment option for patients with CD7-positive tumors who are ineligible for conventional allogeneic HSCT. Additionally, a study conducted by Yue Tan et al. [58] reported the 2-year follow-up of 20 patients with R/R T-ALL treated with CD7 CAR-T cells (ChiCTR2000034762). The results indicated a complete remission rate of 85%, with 35% of patients undergoing stem cell transplantation. Although the relapse rate was 30%, four patients who relapsed lost CD7 expression in their tumor cells. Long-term adverse events included five infections and one instance of grade IV intestinal GvHD. These findings suggest that donor-derived CD7 CAR-T-cell therapy exhibits durable efficacy in some patients with R/R T-ALL, although disease recurrence and severe infections remain significant challenges.

While research continues on donor-derived CAR-T cells, Zhang M and his team [17] focused on autologous-derived CD7 CAR-T cells and conducted preclinical studies using Luc + GFP + CCRF-CEM cells injected into NPG mice. They observed that these CD7 CAR-T cells prevented self-destruction and exhibited potent cytolytic activity, significantly halting leukemia progression and extending mouse survival. In a phase I clinical trial of autologous CD7 CAR-T cells for R/R T-ALL/LBL patients (NCT04004637), 87.5% of participants achieved CR at three months postinfusion, with the majority experiencing only minor side effects. These results indicate that autologously derived CD7 CAR-T cells possess durable and effective therapeutic potential for treating R/R T-ALL/LBL patients with mild side effects.

The preparation of autologous CAR-T cells may face limitations due to the variability in the availability and quality of a patient’s own T cells. Although donor-sourced CAR-T cells can address these issues, they introduce greater complexity and costs, particularly regarding compatibility and the prevention of GvHD. Generic CAR-T cells, which are designed to reduce the risk of GvHD by removing specific immune recognition-related genes, have captured researchers’ interest. Chiesa R and her team [59] utilized gene editing with cytosine deaminase, guided by CRISPR technology, to precisely convert nucleotides—specifically converting cytosine to thymine—without causing DNA breaks. By applying this technique to T cells from a healthy donor, they successfully generated CD7 CAR-T cells (BE-CAR7), achieving initial success in treating three pediatric patients with relapsed leukemia.

Consequently, a research team [18] pursued a novel strategy without gene editing, effectively addressing the problem of self-incompatibility by isolating naturally selected CD7 CAR (NS7CAR) T cells from a vast pool of T cells. In a preliminary clinical trial (NCT04572308) involving 20 R/R T-ALL/LBL patients, 19 achieved bone marrow microscopic residual disease-negative CR, with only mild adverse effects reported, highlighting the excellent outcomes of this therapy. Additionally, as alternative effector cells lacking CD7 expression, NK cells were utilized to develop CD7-redirected CAR-NK cells, which exhibited significant tumor-killing activity. You et al. [19] developed both monovalent and bivalent CD7-directed CAR-NK cells using CD7-specific nanoantibodies and NK-92MI cells. Their findings revealed a significant increase in granzyme B levels and a significant increase in granzyme B and IFN-γ secretion upon targeting CD7-positive primary T-ALL cells, along with the substantial suppression of disease progression in a T-ALL mouse xenograft model. Furthermore, the initiation of two clinical trials (NCT04004637 and NCT04033302), as listed in the National Institutes of Health (NIH) database, represents a critical advance in evaluating the safety and efficacy of CD7 CAR-T cells in treating patients with NKTCL.

### T-cell receptor beta chain constant region (TRBC)

TRBC, encoded by two mutually exclusive genes, TRBC1 and TRBC2, has emerged as an effective target for PTCL therapy. In healthy individuals, TRBC1 and TRBC2 are exclusively expressed in T cells at 25–47%. In contrast, malignant T cells usually express only TRBC1 or TRBC2. Therefore, CAR-T cells targeting TRBC1 specifically eliminate TRBC1-positive malignant T cells while preserving immune-competent TRBC2-positive normal T cells [60]. The TRBC1 CAR-T-cell line developed by Maciocia et al. [21] specifically recognized and eliminated TRBC1-expressing normal and malignant T cells without damaging TRBC2-expressing T cells, resulting in a significant reduction in the tumor load and prolonged survival in T-ALL model mice. In the AUTO4 clinical trial (NCT03590574) led by Cwynarski K and his team [61], the safety and efficacy of an autologous CAR-T-cell therapy targeting TRBC1-positive PTCL were evaluated. At one month posttreatment, of the 10 patients treated, 5 of 9 evaluable patients achieved a complete metabolic response (CMR), 1 achieved a partial response (PR), and 3 experienced no response. Only four patients experienced mild and reversible adverse reactions. These preliminary results indicate an encouraging initial response to AUTO4 treatment. The development of CAR-T-cell therapies targeting TRBC2-positive T cells is also underway [22]. These findings provide novel insights and potential directions for improving the treatment of T-cell malignancies. Although the use of anti-TRBC1 CAR-T cells is a promising approach for the treatment of T-cell malignancies, unsorted T-cell transduction may result in weak or even resistant killing of TRBC1-positive malignant T cells. Zhang et al. [23] reported that in patients lacking sufficient autologous T cells, the use of syngeneic allogeneic T cells for the preparation of anti-TRBC1 CAR-T cells and predepletion of TRBC1-positive T cells is necessary to promote therapeutic efficacy. This information provides an important direction for the further development of TRBC1 CAR-T cells.

### CD2

CD2 is a transmembrane glycoprotein that is highly expressed in NK and T cells (including early progenitor cells) and is essential for T-cell activation and signaling through interactions with its ligands, CD58 (human) and CD48 (mouse) [62]. CD2 expression has been observed in various T-cell malignancies, such as SS, PTCL, and ATLL [63]. Xiang et al. [24] deleted the CD2 and TRAC genes using CRISPR-Cas9 technology to develop CAR-T cells targeting CD2 (CAR2-CD28-CD3ζ), with the goal of targeting and destroying malignant T cells while reducing the risk of GvHD or autoattack. This study also investigated the ability of the combination of this treatment with long-acting recombinant human interleukin-7 (rhIL-7-hyFc) to compensate for the reduced efficacy that may be caused by CD2 deficiency and to improve the persistence and antitumor activity of UCART2 cells. This study provides a new therapeutic direction for targeting T-cell malignancies by effectively circumventing the challenges of autophagy and GvHD through the use of gene editing and cell therapy techniques.

### CD3

The CD3 antigen is ubiquitously present on the surface of all T cells and plays a crucial role in T-cell activation and function. In the treatment of T-cell-derived malignancies, such as PTCL, CD3 shows potential clinical value. However, the widespread expression of CD3 poses challenges in the development of CD3-based CAR-T-cell therapies, especially those with the potential to cause adverse reactions, such as self-incrimination. Innovative strategies have been employed to overcome these problems, such as the use of transcription activator-like effector nuclease (TALEN) technology to disrupt the endogenous TCRαβ/CD3 complex [25] and the utilization of non-T-cell effector cells (e.g., NK-92 cells) as an alternative, which have been effective in reducing adverse reactions and enhancing the specificity and safety of the therapy. Additionally, by precisely controlling the intensity and duration of CD3 signaling in CAR-T cells, an optimal balance between efficacy and safety can be determined. In particular, a study by Cárdenas et al. [26] aimed at optimizing CAR design by modifying the internal signaling domain of CD3 enhanced the efficacy and safety of CAR-T-cell therapy.

### CD4

CD4 + T cells play a critical role in the immune system of healthy individuals. In the two subtypes of CTCL, MF and SS, the clonal expansion of CD4 + T cells highlights their potential as targets for CAR-T-cell therapy [64]. However, CD4 monoclonal antibody treatments against CTCL (e.g., zanolimumab) have shown high response rates in patients with MF but are less effective in patients with SS, indicating significant differences in response to therapy across CTCL subtypes [27]. Initial preclinical studies and phase I clinical trials (NCT03829540, NCT04219319, and NCT04162340) have evaluated the safety, tolerability, and potential efficacy of CD4 + CAR-T cells for the treatment of T-cell malignancies. Studies have also explored NK-92 cells as an alternative therapeutic strategy, given that CD4 is widely expressed on T cells and carries the risk of self-incineration. CD4 CAR-NK-92 cells successfully eliminated malignant T cells and prolonged survival in vitro and in NSG mouse models, indicating their potential for use in the treatment of T-cell malignancies [28].

### CD30

CD30, a member of the tumor necrosis factor receptor family, is expressed predominantly on specific subpopulations of activated T and B lymphocytes and is strongly associated with lymphoma development. It plays a crucial role in cell proliferation and prosurvival signaling in certain PTCL and ATLL subtypes and is particularly highly expressed in specific PTCL types, such as ALCL, where its overexpression is an ideal target for T-cell lymphoma therapies [65]. Nakashima and Uchimaru [29] highlighted the promising clinical outcomes of antibody‒drug conjugates (ADCs) targeting CD30 in managing lymphomas with CD30 overexpression, emphasizing the critical role of CD30 signaling in promoting chromosomal instability, which is crucial for ALCL progression. Additionally, the anti-CD30 drug brentuximab vedotin has proven effective in treating advanced CTCL, although it may lead to increased neurotoxicity [30]. Wu et al. [31] developed CD30 CAR-T cells and demonstrated through cytotoxicity assays and xenograft tumor models that these cells exhibit significant cytotoxicity and suppress CD30-positive tumor cells. Recently, clinical trials exploring CAR-T-cell therapy targeting CD30 for treating patients with R/R CD30 + PTCL have achieved significant results. In a phase I clinical trial (NCT01316146) conducted by Ramos et al. [32], 9 patients with R/R CD30 lymphomas were treated with CD30 CAR-T cells. Half of these patients achieved sustained CR for at least 9 months, specifically those with ALCL, without any impairment to virus-specific immune function. D. Wang et al. treated 9 R/R patients with CD30 CAR-T-cell therapy, including 6 with Hodgkin lymphoma (HL) and 3 with ALCL. Seven patients achieved CR on their first treatment, and three maintained long-term CR for more than two years, with most adverse events being mild and controllable [33]. CD30 CAR-T cells have shown good efficacy and safety; however, their long-term effects and potential side effects on normal cells still require further research. These findings underscore the potential of CD30 as a therapeutic target in T-cell lymphoma and suggest directions for future research. Multiple clinical trials (NCT04083495, NCT04526834, NCT02917083, NCT01316146, NCT03049449, and NCT02690545) are actively exploring CD30 CAR-T-cell therapy in patients with rare R/R CD30-positive PTCL and classical HL, and are expected to drive new advances in the field.

Treatment of enteropathy-associated T-cell lymphoma (EATL), a rare and highly aggressive lymphoma originating from celiac disease, is mainly based on multiagent chemotherapy regimens with anthracyclines; however, the median overall survival is only 7 months, indicating a generally poor prognosis [66]. In patients who respond well to primary treatment and are suitable for autologous stem cell transplantation, the 5-year survival rate can improve by 50–60% [67]. Because most patients with EATL express CD30, brentuximab vedotin, a targeted therapy against CD30, shows promise [68]. In a phase I/II study completed by Voorhees et al. [69], a patient with EATL who experienced multiple relapses achieved durable remission after treatment with CD30 CAR-T cells, revealing the safety and efficacy of CD30 CAR-T cells in treating CD30-positive HL patients.

### CC chemokine receptor 4 (CCR4)

CCR4, a G protein-coupled receptor with seven transmembrane structural domains, regulates T-cell migration and function by binding to its ligands CCL17/TARC and CCL22/MDC. In normal immune responses, CCR4 is mainly expressed in type 2 and 17 helper T cells (Th2 and Th17) and regulatory T cells (Tregs), with low expression in other helper T cells and CD8 + T cells. Given the high expression of CCR4 in a variety of T-cell malignancies, especially MF and SS, which promote the migration of malignant T cells into the skin and are closely associated with disease progression, CCR4 has emerged as a potential therapeutic target [70]. Mogamulizumab, a defossilized humanized anti-CCR4 monoclonal antibody, has been shown to effectively treat T-cell lymphomas by enhancing antibody-dependent cell-mediated cytotoxicity (ADCC) [34]. This drug reduces the number of CCR4 + Tregs, which are important for enhancing the immune response. Based on this finding, Perera et al. [35] developed CAR-T cells targeting CCR4 by specifically depleting Th2 and Tregs while preserving CD8 + and Th1 T cells, reporting significant antitumor effects and long-term remission potential in a mouse model of T-cell lymphoma. These findings suggest that CCR4 is an effective target for the treatment of T-cell malignancies and provides a new strategy for targeting specific immune cell subsets.

### CD26

CD26 is a key membrane surface glycoprotein that is highly expressed, especially in CD4 + T cells, highlighting its importance in antitumor activity, and it has become a promising target for cancer therapy [71]. Kobayashi et al. [36] developed second-generation CD28 costimulatory domain-containing and third-generation CD26-targeted CAR-T cells (CD26-2G/3G) containing the CD28 and 4-1BB costimulatory domains to explore their potential against PTCL. In vitro experiments revealed that CD26-2G/3G CAR-T cells exhibited significant antitumor activity against the T-cell leukemia cell line HSB2. In an in vivo mouse model, CD26-3G CAR-T cells exhibited a greater ability to inhibit tumor growth and improve survival than CD26-2G cells. This study not only confirms that CD26 is an effective target for the treatment of T-cell malignancies but also highlights the potential of CAR-T-cell therapies employing different costimulatory domains to enhance therapeutic efficacy, especially the remarkable anticancer efficacy of third-generation CAR-T-cell therapies, which provides important insights into understanding the mechanism and optimization of therapeutic regimens for CAR-T-cell therapies and provides a new direction for future therapeutic strategies.

### CD70

CD70, a type 2 transmembrane glycoprotein belonging to the tumor necrosis factor (TNF) ligand family, was found to be highly expressed in malignant hematopoietic stem cells and solid tumors and constitutively expressed in activated T-cell leukemias and lymphomas in previous studies, indicating its potential as an immunotherapeutic target [72]. In the COBALT-LYM study (NCT04502446) [37], a CAR-T-cell product (CTX130) targeting CD70 was developed to edit TRAC, β2-microglobulin, and CD70 using CRISPR-Cas9 technology to reduce CAR-T-cell self-interaction and improve therapeutic safety and efficacy. A preliminary clinical trial showed an overall remission rate of 71% and a CR rate of 29% in 15 patients with R/R PTCL, with no dose-limiting toxicities observed. This trial confirmed the efficacy and safety of CAR-T-cell therapy targeting CD70 for the treatment of T-cell tumors, demonstrating the potential of gene editing technology for optimizing CAR-T-cell therapy.

### CD276 (B7-H3)

B7 homolog 3 (B7-H3), also known as CD276, is a member of the B7 family of immunomodulatory proteins that is overexpressed in a variety of solid tumors and is associated with disease progression, an increased risk of recurrence, decreased survival, and drug resistance, making it a potential target for cancer immunotherapy [73]. In a study of ALCL cell lines derived from clinical samples and ALK-induced T-cell transformation models, Zi et al. [38] developed B7-H3-specific CAR-T cells. These cells exhibited significant cytotoxicity against ALCL cell lines in vitro and successfully cleared ALCL from a mouse model in vivo, providing a scientific basis for further research and development of B7-H3 CAR-T cells for ALCL therapy. Further studies showed that the effectiveness of B7-H3 CAR-T-cell therapy was closely related to the expression density of the target antigen B7-H3 on the surface of tumor cells. This finding underscores the importance of monitoring the level of B7-H3 expression on the tumor surface during B7-H3 CAR-T-cell therapy and accordingly suggests a drug management strategy aimed at improving treatment safety. The precise adjustment of the treatment regimen aims to maximize efficacy while reducing potential adverse effects, providing patients with safer and more effective treatment options [74].

NKTCL is a rare and highly aggressive subtype of NHL closely related to EBV infection and is characterized by extranodal involvement. NKTCL cells are resistant to anthracycline-containing drugs, which is partly due to their high expression of P-glycoprotein. Despite the recommendation of the ork NCCN guidelines for the use of L-asparaginase-based treatment regimens, the survival of patients with R/R NKTCL remains suboptimal. For NKTCL, CAR-T cells targeting B7-H3 have shown potent cytotoxicity in mouse models [75]. Considering the close association between NKTCL and EBV infection, latent membrane protein 1 (LMP1) is also considered a potential therapeutic target [76]. In a preclinical study, Li et al. [77] prepared four CAR-T-cell lines (CD38-CAR, LMP1-CAR, and CD38-LMP1 tandem CARs 1 and 2) and evaluated their effects on NKTCL cells. These CAR-T-cell lines effectively eliminated malignant NKTCL cells and significantly inhibited tumor growth in a mouse NKTCL xenograft model, indicating their potential for the effective treatment of NKTCL.

Extranodal nasal NK/T-cell lymphoma (ENKTCL) is another rare and highly aggressive subtype of NHL with a low 5-year overall survival rate, and novel treatment strategies are urgently needed. Immunotherapeutic advances, including CAR-T cells and bispecific T-cell binding (BiTE) antibodies, represent new directions for the treatment of this type of tumor. Zheng et al. [78] revealed for the first time the highly homogeneous expression of B7-H3 in ENKTCL and developed BiTE antibodies and CAR-T cells against B7-H3. These novel therapeutic strategies displayed effective targeting and killing ability against NKTCL cells in vitro and in vivo and inhibited the growth of NKTCL tumors in the NSG mouse model, suggesting that B7-H3 is a promising target for NKTCL therapy.

### CD37

CD37, a transmembrane protein, is a member of the transmembrane 4 superfamily and is expressed mainly in mature B cells but also at low levels in T cells, NK cells, and some monocytes [79]. AGS67E, an antibody‒drug coupling agent against CD37, has shown partial efficacy and a favorable safety profile in clinical trials for the treatment of CTCL [39]. The CD37 CAR-T cells developed by Scarfo’s team for CTCL were shown to specifically activate and secrete cytokines to effectively kill CTCL cell lines from patients (e.g., HuT78) in in vitro experiments with no apparent T-cell self-interaction [40]. Due to the weak expression of CD37 in normal T cells, CAR-T cells targeting CD37 for the treatment of T-cell lymphomas are expected to minimize the risk of autophagy and impaired T-cell regeneration. A phase I clinical trial of CD37 CAR-T-cell therapy is currently underway (NCT04136275), indicating the potential of CD37 as a therapeutic target in the treatment of T-cell lymphoma.

### CD47

CD47, an integrin-associated protein widely expressed on nucleated cells and erythrocytes, inhibits phagocytosis by binding to signal-regulated protein α (SIRPα) on macrophages, transmitting a “don’t eat me” signal that helps hematological and solid tumors evade immune surveillance [80, 81]. CD47 is highly expressed in peripheral blood and skin Sézary cells in SS, and its level is negatively correlated with overall patient survival [41]. The use of SIRPαFc (TTI-621), a decoy receptor that blocks the interaction of CD47 with SIRPα, was shown to provide a therapeutic benefit to patients with SS by significantly reducing the tumor load through the inhibition of the CD47-SIRPα signaling pathway in a clinical trial (NCT02663518). Thus, CD47 is a potential target for the treatment of SS through immune escape strategies and warrants further exploration and development.

### B7-H6

B7-H6, a novel costimulatory molecule, is expressed only in a wide range of tumor cells and is correlated with a poor prognosis. In T-LBL, B7-H6 expression is significantly associated with several poor prognostic factors, such as B symptoms, a high Eastern Cooperative Oncology Group (ECOG) score, elevated serum lactate dehydrogenase (LDH) levels, and a poor response to therapy, suggesting its importance as a therapeutic target [42]. Yuan et al. [42] reported that 61.5% of 65 T-LBL samples expressed B7-H6, with 38.5% of the patients expressing B7-H6 in both the cell membrane and cytoplasm. Experiments depleting B7-H6 in Jurkat cells showed that B7-H6 was involved in the proliferation, migration, and invasion of T-LBL cells, providing novel insights into the biological properties of T-LBL and the development of new therapeutic strategies. Future studies are required to further explore the role and therapeutic potential of B7-H6 in T-LBL, to develop effective therapeutic approaches, and to improve the prognosis of patients with T-LBL.

### Cell division cycle 27 (CDC27)

CDC27 is a key cell cycle protein that is essential for mitotic progression and is an important component of the late-promoting complex/cyclosome (APC/C). Its aberrant expression is closely associated with the development of several cancers [82]. In particular, CDC27 overexpression in T-LBL is associated with disease progression and reduced patient survival, highlighting its importance in the pathogenesis of T-LBL and providing clues for the discovery of new therapeutic targets [43]. CDC27 promotes T-LBL cell proliferation, inhibits apoptosis, and is positively correlated with the expression of programmed death ligand-1 (PD-L1), which acts as a ligand for the immune checkpoint PD-1. Overexpression of PD-L1 in multiple tumors is correlated with immune escape [44]. Therefore, the association between CDC27 and PD-L1 provides new research directions for T-LBL immunotherapy, particularly for checkpoint inhibitors targeting the PD-1/PD-L1 axis.

### T-cell receptor (TCR) β-chain (TCRVβ) variants

In response to the therapeutic challenges of ATLL, researchers have explored therapeutic strategies targeting TCRVβ, the β-chain in the cell-surface TCR α/β-chain heterodimer, a structure that is specifically present on normal and malignant T cells. Targeting the T-cell lymphoma-specific TCRVβ subunit provides a highly selective and specific therapeutic strategy capable of precisely eliminating tumor cells while minimizing the toxic effects on healthy T cells [83]. The anti-TCRVβ CAR-iNKT cell therapy strategy developed by Rowan et al. [45] revealed its specificity and efficacy in targeting T-cell lymphoma cells. This engineered iNKT cell therapy not only avoids the risk of GvHD but also provides new hope for the treatment of refractory T-cell malignancies, such as ATLL, by effectively inhibiting T-cell malignant tumor growth in vivo as an immediately available immunotherapy that is highly selective for malignant cells in patients with ATLL.

### CCR8

The chemokine receptor CCR8 is a G protein-coupled receptor that is regulated mainly by Chemokine CCL1. CCR8 is highly expressed in the tumor microenvironment, especially in Tregs, whereas NK cells, CD8 + T cells, myeloid cells, and most CD4 + T cells do not express CCR8. This unique expression pattern makes CCR8 a potential target for immunotherapy. In particular, blockade of CCR8 reduces the number of Tregs in the tumor microenvironment, thereby enhancing antitumor immune responses [84]. Zheng et al. [46] reported a significant increase in CCR8 expression in an ATLL model and showed that the CAR-T cells targeting CCR8 that they developed exhibited potent antitumor activity that could be achieved without affecting normal T-cell expansion and without impairing the immunosuppressive function of Treg cells to prolong the survival of tumor-bearing mice. This study identified CCR8 as a new CAR-T-cell therapeutic target, providing a novel strategy for the treatment of tumors, such as ATLL.

## Fratricide

**Fig. 2:**
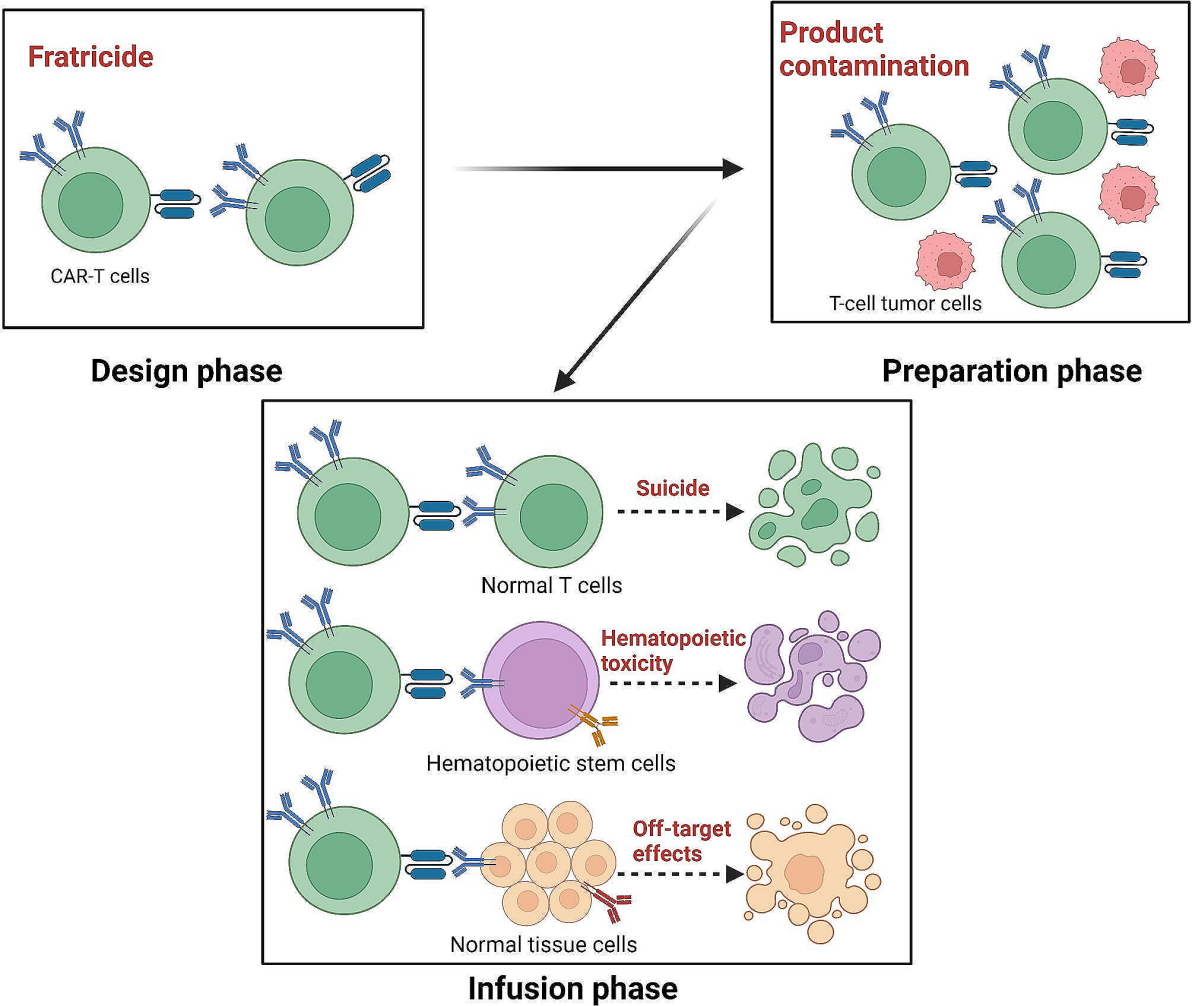
Challenges faced by CAR-T-cell therapy in the treatment of T-cell malignancies. In the design phase of CAR-T-cell therapy, the sharing of target antigens may trigger self-incrimination. At the preparation stage, malignant T cells may be mixed into the CAR-T-cell product, leading to contamination. CAR-T cells infused into patients may cause suicidal effects by killing normal T cells, and the killing of hematopoietic stem cells may lead to hematopoietic toxicity; moreover, the accidental killing of normal tissue cells may cause off-target effects. Created with BioRender.com

During the development of CAR-T-cell therapies for T-cell malignancies, researchers have encountered a number of unique and complex challenges that are significantly different from those of CAR-T-cell therapies for B-cell malignancies. One of the most critical issues is the phenomenon of “target antigen sharing,” whereby antigenic targets on the surface of T cells are not only expressed on malignant T cells but also are prevalent on normal T cells and on the CAR-T cells themselves. The challenges of CAR-T-cell therapy for the treatment of T-cell malignancies are shown in Fig. 2. During CAR-T-cell production, the self-expression of target antigens can lead to the self-attack of CAR-T cells, a process known as fratricide. This process generally occurs during the in vitro culture and expansion stages and represents a major challenge in developing CAR-T-cell therapies for T-cell malignancies [85].

Researchers have employed various strategies, including gene editing to knock out or downregulate specific target antigens, such as CD7 and CD5, on CAR-T cells to combat fratricide. This approach aims to diminish mutual recognition and subsequent attack among T cells. Additionally, researchers have developed bispecific or multispecific chimeric antigen receptors (CARs) with “logic gating” mechanisms. These designs require the simultaneous recognition of multiple antigens to activate T cells, effectively reducing mistargeting and decreasing the risk of fratricide [86, 87]. Furthermore, researchers are identifying target antigens exclusively expressed on malignant cells rather than on normal or CAR-T cells, enhancing the specificity and safety of CAR-T-cell therapies. For instance, CAR-T cells targeting antigens such as CD30, CD70, and CD276 (B7-H3), which are primarily expressed in specific T-cell subsets, or CD37, CD47, and B7-H6, which are seldom, if ever, expressed in T cells, have shown minimal self-inflicted killing.

In addition to targeting specific antigens, researchers have developed several new strategies to mitigate self-incompatibility. In advanced immunotherapy applications, double-negative T cells (DNTs) have shown robust resistance and safety against tumor cells in in vitro studies due to their lack of CD4 and CD8 expression. Specifically, DNTs engineered with CD19 CAR exhibited safe and potent cytotoxicity against CD19-positive B-cell tumors [88]. A study by Fang et al. [89] further investigated the cytotoxic effects of CD4 CAR-engineered DNTs against T-ALL and PTCL. Given that DNTs inherently do not express CD4, this design circumvents self-inflicted damage and enhances treatment safety. Similarly, CAR-NK cells, which serve as an effective complement to CAR-T-cell therapy, exhibit significant potential in treating T-cell malignancies. NK cells effectively sidestep the issue of self-destruction due to their lack of specific T-cell target antigens [90]. Recent studies have also verified the therapeutic potential of modified CD5 CAR-NK cells, as well as CAR-NK cells targeting CD4 and CD7, in ex vivo experiments [19, 28, 50]. These advancements not only highlight the significant potential of CAR-NK cell therapy but also suggest new avenues for future therapeutic strategies, particularly in addressing the limitations of existing CAR-T-cell therapies. Although these innovative strategies provide effective solutions to the problem of autophagy, their long-term effects and safety must still be validated in future clinical trials.

## Product contamination

In CAR-T-cell therapy, T cells are genetically engineered with CARs, enabling them to specifically recognize and attack cancer cells. The therapeutic process involves several steps: harvesting (extraction of T cells from the patient), genetic engineering (insertion of the CAR gene), expansion (propagation of CAR-T cells in the laboratory), and reinfusion (injection of the modified cells into the patient). Once in the body, CAR-T cells recognize and destroy targeted cancer cells. Their prolonged presence allows them to continuously monitor and eliminate any recurring cancer cells. When transitioning from preclinical research to clinical application, CAR-T-cell therapies encounter significant challenges, including the scalability of production, logistics, and cost-effectiveness. Technical and administrative hurdles impede large-scale production due to the need for customization of each patient’s treatment. The treatment process requires a tight timeline from cell collection to reinfusion, necessitating efficient logistics to ensure cell viability and safety. Furthermore, the high cost of CAR-T-cell therapy, involving advanced equipment, specialized procedures, and costly gene editing technologies, significantly restricts its widespread adoption.

In treating T-cell malignancies, autologous CAR-T-cell therapy must differentiate between healthy and malignant T cells to prevent the contamination of CAR-T-cell products with malignant cells. However, the absence of distinct markers causes the mixing of healthy and malignant T cells during collection and preparation, which can interfere with CAR-T-cell function and potentially exacerbate the disease [91]. Utilizing specific non-disease-associated T-cell subsets, such as CD8 + T cells, to prepare CAR-T cells may enhance therapeutic outcomes. Particularly when battling certain tumor types, CAR-T cells derived from CD8 + T cells are crucial due to their robust tumor-fighting capabilities and enduring immune memory [92]. However, effectively balancing the ratio of CD4 + to CD8 + CAR-T cells to optimize therapeutic benefits requires further in-depth study. Additionally, the use of allogeneic CAR-T cells (allo-CAR-T cells) from healthy donors represents a new therapeutic option for treating T-cell malignancies. While this approach holds promise for treating T-cell malignancies, potential side effects, such as GvHD, must be carefully considered [93]. Both autologous and allogeneic CAR-T cells have unique therapeutic potential, each with distinct benefits and challenges. A phase I clinical trial by Zhang [94] assessed the safety and efficacy of anti-CD7 CAR-T cells in treating adolescents and adults with R/R T-cell malignancies and revealed that patients treated with allogeneic CAR-T cells achieved a significantly higher CR rate than those treated with autologous CAR-T cells and exhibited lower relapse rates and prolonged CAR-T-cell survival. Generic CAR-T cells engineered to reduce the risk of GvHD by removing specific immune recognition-related genes were initially successful in treating three pediatric patients with relapsed leukemia [59]. Given the small sample size and the study’s limited adjustment for disease variability, these preliminary results must be validated further through additional research and extended follow-up. Overall, selecting the type of CAR-T-cell therapy involves considering a combination of patient-specific circumstances, treatment urgency, available medical resources, and anticipated risks. Autologous CAR-T cells provide a relatively safe, albeit sometimes limited, option; donor-derived CAR-T cells broaden treatment applicability but with increased risks; and universal CAR-T cells, potentially the future of therapy, promise greater flexibility and cost-effectiveness, although their long-term safety and efficacy have yet to be established.

## Adverse reactions after CAR-T-cell transfusion

### Suicide

Due to target antigen sharing, CAR-T cells attack not only their counterparts during preparation—a process known as fratricide—but also all normal T cells expressing the same antigen postinfusion, a phenomenon referred to as “suicide.” This process severely impairs normal T-cell function, leading to significant immunodeficiency and an increased risk of fatal infections [95]. Researchers are focusing on targets not found on T cells, such as CD37, CD47, and B7-H6, to minimize damage to healthy cells and reduce the risk of serious adverse effects. These targets are minimally or not expressed on T cells, effectively eliminating the risk of suicidality. Additionally, scientists are employing CRISPR-Cas9 gene editing to remove the endogenous TCR and the immune checkpoint protein PD-1. This approach enhances the CAR-T-cell tumor recognition and attack capabilities while reducing the risk of autoimmune-triggered toxicity [96, 97]. Researchers have also discovered that knocking down both PD-1 and CTLA-4 inhibitory receptors in T cells generates stronger antitumor responses [98].

Cytokines, known for promoting T-cell survival and proliferation, are employed to enhance the functionality and persistence of CAR-T cells. Xiang et al. [24] addressed the potential reduced efficacy of CD2 deletion by developing CD2 CAR-T cells without surface CD2 and incorporating IL-7. This approach not only enhanced persistence and antitumor activity but also effectively mitigated issues such as suicide and GvHD. Additionally, the CD5 CAR-T cells developed by Jia Feng et al. utilized soluble IL-15 to enhance malignant T-cell clearance and simultaneously protect normal T cells. Other researchers [15] utilized soluble IL-15 to enhance malignant T-cell clearance and simultaneously protect normal T cells. Another mitigation strategy involves incomplete CAR transduction during the manufacturing of infused CAR-T cells. The dosage of commercialized CAR-T-cell products correlates with the number of viable CAR-expressing (“CAR-positive”) cells. γ-Retroviral and lentiviral manufacturing methods yield approximately 60–90% and 20–60% CAR-positive cells, respectively [99]. Even those cells expressing a lower percentage of CAR can still provide essential immune functions, thus enhancing efficacy and reducing side effects.

Moreover, implementing a switch mechanism is crucial when patients experience severe infections and significant impairments in T-cell function; deactivating CAR-T-cell function can restore normal T-cell activity [100]. The herpes simplex virus thymidine kinase (HSV-TK) with ganciclovir (GCV) system acts as a powerful suicide switch, converting GCV into its active form, which integrates into DNA and causes strand termination and cell death [101]. In the TK007 clinical trial [102], T cells modified with this system showed no acute toxic response, were sensitive to GCV, and effectively controlled GvHD. Given the potential of this technology to control GvHD, researchers propose that its application in CAR-T cells could yield similar benefits, necessitating further investigation and development. In addition, the inducible cysteine asparaginase-9 (iCasp9) suicide gene strategy activates the cell’s endogenous apoptotic pathway via iCasp9, swiftly inducing cell death. This approach offers an added safeguard in CAR-T-cell therapy, swiftly eliminating overactivated or unresponsive CAR-T cells [103]. Additionally, CD20, a surface protein primarily found on B cells, is targeted and cleared by rituximab, which induces complement-dependent cytotoxicity (CDC). Researchers have introduced CD20 into T cells as a suicide gene, enabling cell elimination via rituximab-mediated killing, thus enhancing the safety and controllability of adoptive T-cell therapies [104]. The expression of CD20 on transduced T cells enables their clearance by rituximab when necessary, such as in cases of severe GvHD, thus enhancing the safety of the therapy [105]. Additionally, Philip B and his team developed RQR8 [106], a compact marker/suicide gene that combines CD34 and CD20 antigenic epitopes. By merging marking and suicide functionalities, RQR8 facilitates cellular selection and elimination using the clinically approved CliniMACS CD34 system and rituximab. This CD20-labeled T-cell technology has potential future applications in preparing CAR-T cells, enhancing their safety for clinical use. However, CRISPR-Cas9-based CAR-T-cell therapy continues to face safety challenges, including off-target effects and the risk of chromosomal deletions, potentially impacting the genetic stability of modified T cells. Its long-term efficacy and safety require further investigation and validation in multiple clinical trials [107].

In addition to gene editing, nongene editing approaches such as protein expression blockers (PEBLs) are employed to inhibit the binding of target antigens to CAR single-chain variable fragments (scFv). This approach not only preserves the proliferative capacity of CAR-T cells but also aids in preventing GvHD [108]. The SNIP-CARs technology, a protease-regulated platform, utilizes small-molecule drugs such as grazoprevir to modulate the activity of CAR-T cells. This strategy maintains CAR-T cells in an inactive state until they are reactivated by a drug, indicating its potential as a therapeutic safety switch [109].

If T-cell suicide occurs, the resulting immunodeficiency may be mitigated by using CAR-T-cell therapy as a bridge to allogeneic hematopoietic stem cell transplantation (allo-HSCT), which aids in immune reconstitution and reduces the risk of aplastic disorders [53]. Li [110] proposed the use of donor-derived CD7 CAR-T cells as a novel pretreatment strategy to bridge allogeneic allo-HSCT, offering potential therapeutic improvements. This approach not only controls infections and improves disease-free survival but also boosts the therapeutic efficacy of CAR-T cells by simplifying the collection process and reducing the risks associated with harvesting patient-derived T cells. Allogeneic HSCT supplies new, healthy hematopoietic stem cells that can rebuild the patient’s immune system and help eliminate any residual malignant cells. Moreover, CAR-iNKT cell therapy targeting TCRVβ has shown specificity and efficacy in attacking T-cell malignancies while preserving healthy T cells [82].

In summary, faced with challenges such as suicide and immunosuppression from CAR-T-cell therapies, scientists are continuously refining therapeutic approaches to enhance safety and efficacy, leveraging both innovative gene editing and nongene editing strategies.

### Hematopoietic toxicity

Hematopoietic toxicity from CAR-T-cell therapy often results from “normal T-cell suicide”, which is triggered by the presence of target antigens on T cells that are also expressed on hematopoietic stem cells or other bone marrow cells. This process disrupts the bone marrow environment and impairs blood cell production. Clinically, this toxicity manifests as increased infection risks due to leukopenia, fatigue and pallor from anemia, and increased bleeding risks due to thrombocytopenia. Treatment typically involves closely monitoring the patient and providing supportive care, including prophylactic antibiotics, blood transfusions, and platelet transfusions [111, 112]. Targets widely expressed in hematopoietic tissues like CD5, CD7, CD2, CD3, and CD4, particularly CD7, which is found on hematopoietic stem cells, pose significant therapeutic risks. CAR-T cells targeting these antigens can increase the risk of myelosuppression at administration, necessitating a careful evaluation of target selection to minimize off-target effects, particularly concerning antigens present in hematopoietic cells. Furthermore, researchers are developing more selective CAR-T-cell designs, such as dual-target or multitarget CAR-T cells, requiring the simultaneous recognition of multiple targets for activation. This approach aims to reduce off-target effects and mitigate hematopoietic toxicity [86, 87]. This research is crucial for enhancing the safety and efficacy of CAR-T-cell therapies, ensuring the maximum protection of normal cells and minimizing side effects while effectively targeting and destroying cancer cells.

### Other adverse reactions

In CAR-T-cell therapy for T-cell malignancies, off-target effects occur when selected CAR target antigens are expressed on normal cells, leading CAR-T cells to mistakenly attack these nontumor cells. Antigens such as B7-H6, CD30, CD70, and CD276 (B7-H3) are highly expressed on tumor cells but are also found on normal cells, thereby increasing the risk of nontumor cell targeting. CD26, which is widely expressed in the digestive and respiratory systems, can lead to severe inflammation in these areas when targeted by CAR-T-cell therapy. CCR4 is primarily expressed in regulatory T cells, and targeting this antigen may disrupt immune system regulation. Managing these complications typically involves a combination of prophylactic antibiotics, corticosteroids to mitigate inflammation, and supportive therapies such as blood or platelet transfusions for bleeding tendencies. GvHD occurs when CAR-T cells attack a patient’s normal tissues, typically resulting in multiorgan damage [113]. For instance, CAR-T cells targeting CD47 may inadvertently attack normal tissues because of its widespread expression, potentially causing GvHD. Common symptoms of GvHD include skin rash, abnormal liver function, and gastrointestinal issues. The treatment of GvHD depends on its severity; mild cases may be managed with basic immunosuppressive therapy [114], while severe cases necessitate more intensive immunosuppressive treatments to prevent long-term tissue damage [115]. CRS is a systemic inflammatory response triggered by cytokines released from CAR-T cells. The initial symptoms, which typically appear within the first two weeks, include fever and vomiting. Patients with severe CRS may experience life-threatening hypoxemia and respiratory failure [116]. The severity of CRS is linked to the number of proliferating CAR-T cells infused and the patient’s tumor burden [117]. Low-grade CRS is managed symptomatically with corticosteroids, while high-grade CRS requires both corticosteroids and IL-5 receptor antagonists such as tocilizumab. Immune effector cell-associated neurotoxicity syndrome (ICANS) is an adverse reaction to CAR-T-cell therapy characterized by changes in the CNS caused by activated infused T cells. This syndrome can present with symptoms such as cognitive dysfunction and coma. Secondary cerebral edema, a critical factor contributing to its severity, should be treated with dexamethasone or methylprednisolone, which can penetrate the blood‒brain barrier [118].

## Conclusions

CAR-T-cell therapy has shown significant potential in treating T-cell malignancies, although this technology remains in the early stages of research. Given the complexity and therapeutic challenges of treating T-cell malignancies, such as tumor heterogeneity, resistance to chemotherapy, and a poor prognosis, researchers are actively exploring innovative strategies to optimize treatment efficacy. In CAR-T-cell therapy for T-cell malignancies, target selection is a key factor. Currently, in addition to the commonly utilized targets CD5 and CD7, researchers have identified additional effective CAR-T-cell targets for treating T-cell malignancies, including CD30, CD26, CD70, CCR4, and CD276 (B7-H3). Although these targets inevitably exhibit suicidal effects when infused into patients, they significantly mitigate the risk of fratricide during CAR-T-cell preparation. Moreover, targets such as CD37, CD47, B7-H6, CDC27, TCRVβ, and CCR8, when employed in CAR-T cells, present minimal risks of fratricide and autoimmunity. The sharing of target antigens poses a major challenge in CAR-T-cell preparation, potentially leading to self-incineration and significantly reduced production efficiency. Researchers have utilized gene editing techniques to knock out or down-regulate specific target antigens on CAR-T cells, such as CD7 and CD5, to address this issue, thereby reducing mutual recognition and attack among T cells. Additionally, researchers have developed bispecific or multispecific CAR designs featuring “logic gating” mechanisms that more accurately distinguish malignant cells from normal cells, enhancing the specificity and safety of CAR-T cells. Furthermore, the use of DNTs, NK cells, and allogeneic T cells provides new effector cell vectors for therapies capable of circumventing autologous and suicidal phenomena under specific circumstances. Product contamination of CAR-T cells prior to preparation and infusion remains a significant challenge, particularly due to potential interference in CAR-T-cell function from shared target antigens. Investigators have utilized specific subpopulations of nondisease-associated T cells and allogeneic CAR-T cells from healthy donors to minimize product contamination, effectively enhancing the safety and feasibility of therapy. Following infusion, CAR-T cells may induce adverse reactions, notably T-cell suicide, leading to severe immunosuppression and an increased risk of fatal infections. Beyond employing innovative gene and nongene editing strategies, these challenges have been addressed using strategies such as allo-HSCT to offer patients new hope for immune recovery and disease control. The challenges and strategies for the use of CAR-T-cell therapy in patients with T-cell malignancies are shown in Table 2.

**Table 2:** Challenges and strategies to address the use of CAR-T-cell therapies in treating T-cell malignancies

Challenges and strategies	Research phases	Efficacy
**Fratricide**		
Knocking out or downregulating specific target antigens of CAR-T cells themselves with gene editing techniques	Preclinical and clinical studies [9–11, 20, 48]	Pending verification
Dual-target or multitarget CAR-T cells	Preclinical and clinical studies [86, 87]	Effective
Targets specifically expressed only on malignant T cells (e.g., CD30, CD70, and CD276)	Preclinical and clinical studies [31–33, 37, 78]	Pending verification
Construction of CAR-T cells with DNTs	Preclinical studies [88, 89]	Pending verification
Constructing CAR-NK cells	Preclinical and clinical studies [19, 28, 50, 90]	Uncertain
**Product Contamination**		
Preparation of CAR-T with non-disease-associated T-cell subsets	Preclinical and clinical studies [92]	Uncertain
Allogeneic CAR-T cells from healthy donors	Clinical studies [93, 94]	Effective
**Suicide**		
Targets specifically expressed only on malignant T cells (e.g., CD30, CD70, and CD276)	Preclinical and clinical studies [31–33, 37, 78]	Pending verification
Removal of endogenous TCR, immune checkpoint protein PD-1, and CTLA-4 inhibitory receptor using gene editing techniques	Preclinical and clinical studies [96–98]	Pending verification
Combined use of cytokines (e.g., IL-7 and IL-15)	Preclinical and clinical studies [15, 20, 24, 41]	Effective
Manufacture of incompletely CAR-transduced CAR-T cells	Preclinical studies [99]	Pending verification
Suicide genes (e.g., HSV-TK) and safety switches (e.g., iCasp9, CD20, and RQR8)	Preclinical and clinical studies [100–107]	Pending verification
Protein expression blockers	Preclinical studies [108, 109]	Uncertain
allo-HSCT	Clinical studies [15, 110]	Effective
Constructing CAR-NK cells	Preclinical and clinical studies [19, 28, 50, 90]	Uncertain
**Hematopoietic toxicity**		
Targets specifically expressed only on malignant T cells (e.g., CD30, CD70, and CD276)	Preclinical and clinical studies [31–33, 37, 78]	Pending verification
Dual-target or multitarget CAR-T cells	Preclinical and clinical studies [86, 87]	Effective
Symptomatic treatment	Clinical studies [111, 112]	Effective
**Other adverse reactions (off-target effects, GvHD, CRS, and ICANS)**		
Comprehensive management (symptomatic treatment, immunosuppressive treatment, and medication)	Clinical studies [111, 112, 114, 118]	Effective

Abbreviations: allo-HSCT: allogeneic hematopoietic stem cell transplantation; CAR-NK cells: chimeric antigen receptor natural killer cells; CAR-T cells: chimeric antigen receptor T cells; CTLA-4: cytotoxic T-lymphocyte-associated protein 4; DNTs: double-negative T cells; HSV-TK: herpes simplex virus thymidine kinase; iCasp9: inducible caspase 9; PD-1: programmed death-1; TCRs: T-cell receptors

In conclusion, despite the numerous challenges faced by CAR-T-cell therapy in treating T-cell malignancies, its future remains promising due to the meticulous screening of tumor-specific antigenic targets, the application of gene editing techniques, and the exploration of new cellular vectors, as well as ongoing research and clinical trials. This approach not only offers hope for a cure for patients with T-cell malignancies but also advances the entire field of tumor therapy, enhancing treatment possibilities and improving the quality of life for future cancer patients. Future research will concentrate on overcoming the existing challenges and advancing the development of CAR-T-cell therapy for the treatment of T-cell malignancies.

## Data Availability

No datasets were generated or analysed during the current study.

## Abbreviations

ADCC: Antibody-dependent cell-mediated cytotoxicity
ADCs: Antibody‒drug conjugates
AITL: Angioimmunoblastic T-cell lymphoma
ALCL: Mesenchymal anaplastic large cell lymphoma
allo-HSCT: Allogeneic hematopoietic stem cell transplantation
ATLL: Adult T-cell leukemia/lymphoma
BiTE: Bispecific T-cell binding
CAR-T: Chimeric antigen receptor T-cell
CARs: Chimeric antigen receptors
CCR4: CC chemokine receptor 4
CDC27: Cell division cycle 27
CDC: Complement-dependent cytotoxicity
CMR: Complete metabolic response
CNS: Central nervous system
CR: Complete remission
CRS: Cytokine release syndrome
CTCL: Cutaneous T-cell lymphoma
DNTs: Double-negative T cells
EATL: Enteropathy-associated T-cell lymphoma
EBV: Epstein‒Barr virus
ENKTCL: Extranodal nasal natural killer/T-cell lymphoma
FDA: Food and Drug Administration
GCV: Ganciclovir
GvHD: Graft-versus-host disease
HL: Hodgkin lymphoma
HSCT: Hematopoietic stem cell transplantation
HSV-TK: Herpes simplex virus thymidine kinase
HTLV-1: Human T-cell leukemia virus type 1
ICANS: Immune effector cell-associated neurotoxicity syndrome
iCasp9: Inducible cysteine asparaginase-9
IVIG: Intravenous immunoglobulin
LMP1: Latent membrane protein 1
MF: Mycosis fungoides
NHLs: Non-Hodgkin lymphomas
NIH: National Institutes of Health
NK: Natural killer
NKTCL: Natural killer (NK)/T-cell lymphoma
PD-L1: Programmed death ligand-1
PEBLs: Protein expression blockers
PR: Partial response
PTCL-NOS: PTCL, not otherwise specified
PTCL: Peripheral T-cell lymphoma
R/R: Relapsed/refractory
scFv: Single-chain variable fragment
SIRPα: Signal-regulated proteinα
SS: Sézary syndrome
T-ALL/LBL: T-lymphocytic leukemia/lymphoma
TALEN: Transcription activator-like effector nuclease
TCR: T-cell receptor
TCRVβ: T-cell receptor β-chain
TNF: Tumor necrosis factor
TRBC: T-cell receptor beta chain constant region
Tregs: Regulatory T cells
